# Hypoxia Selectively Impairs CAR-T Cells In Vitro

**DOI:** 10.3390/cancers11050602

**Published:** 2019-04-30

**Authors:** Robert Berahovich, Xianghong Liu, Hua Zhou, Elias Tsadik, Shirley Xu, Vita Golubovskaya, Lijun Wu

**Affiliations:** 1 ProMab Biotechnologies, 2600 Hilltop Drive, Richmond, CA 94806, USA; robert.berahovich@promab.com (R.B.); Xianghong.liu@promab.com (X.L.); hua.zhou@promab.com (H.Z.); elias.tsadik@promab.com (E.T.); shirley.xu@promab.com (S.X.); 2 Department of Medicine, University of Oklahoma, Health Sciences Center, Oklahoma City, OK 73104, USA

**Keywords:** CAR-T, hypoxia, tumor, microenvironment, CD19, BCMA, immunotherapy

## Abstract

Hypoxia is a major characteristic of the solid tumor microenvironment. To understand how chimeric antigen receptor-T cells (CAR-T cells) function in hypoxic conditions, we characterized CD19-specific and BCMA-specific human CAR-T cells generated in atmospheric (18% oxygen) and hypoxic (1% oxygen) culture for expansion, differentiation status, and CD4:CD8 ratio. CAR-T cells expanded to a much lower extent in 1% oxygen than in 18% oxygen. Hypoxic CAR-T cells also had a less differentiated phenotype and a higher CD4:CD8 ratio than atmospheric CAR-T cells. CAR-T cells were then added to antigen-positive and antigen-negative tumor cell lines at the same or lower oxygen level and characterized for cytotoxicity, cytokine and granzyme B secretion, and PD-1 upregulation. Atmospheric and hypoxic CAR-T cells exhibited comparable cytolytic activity and PD-1 upregulation; however, cytokine production and granzyme B release were greatly decreased in 1% oxygen, even when the CAR-T cells were generated in atmospheric culture. Together, these data show that at solid tumor oxygen levels, CAR-T cells are impaired in expansion, differentiation and cytokine production. These effects may contribute to the inability of CAR-T cells to eradicate solid tumors seen in many patients.

## 1. Introduction

Autologous chimeric antigen receptor (CAR) T cells specific for CD19 provide a substantial therapeutic benefit for a large percentage of patients with B cell leukemias and lymphomas [1,2,3]. Two types of CD19-specific CAR-T cells, tisagenlecleucel (Kymriah) and axicabtagene ciloleucel (Yescarta), have been approved for clinical use by the FDA [4]. Unlike B cell-specific CAR-T cells, CAR-T cells specific for antigens on solid tumors have to overcome multiple immunosuppressive mechanisms intrinsic to the tumor microenvironment [5,6,7,8]. Solid tumors are hypoxic (1% oxygen or less), contain high levels of soluble factors like TGF-β that directly inhibit T cell function [9,10], contain immunosuppressive myeloid cells and regulatory T cells [11,12], and express ligands for checkpoint proteins like PD-1 that down-regulate T cell function [13,14]. Each of these mechanisms have been studied using tumor-specific T cells, but little is known about how these mechanisms affect CAR-T cells.

Hypoxia is also present in bone marrow hematopoietic niches where B lineage cells reside [15]. Therefore, in this study, we analyzed the effects of hypoxia on CD19 CAR-T cells [16,17] and B cell maturation antigen (BCMA) CAR-T cells in vitro [18]. We generated CD19 and BCMA CAR-T cells at atmospheric (18%) and hypoxic (1%) oxygen levels, and characterized the cells for expansion, CAR expression, CD4:CD8 ratio and differentiation status. We then cultured the CAR-T cells with antigen-positive and antigen-negative tumor cells at the same or lower oxygen level, and measured CAR-T cell cytotoxicity, cytokine production and PD-1 upregulation. The data show that both CD19 and BCMA CAR-T cells are not impaired by hypoxia with regards to CAR expression, cytotoxicity or PD-1 expression. However, hypoxia reduces CAR-T cell expansion and differentiation, increases the CD4:CD8 ratio, and substantially reduces cytokine and granzyme B production. These data are critical for the development of next-generation CAR-T cells against tumors with hypoxic microenvironments.

## 2. Results

### 2.1. Hypoxia Greatly Decreases CAR-T Cell Expansion

**Figure 1:**
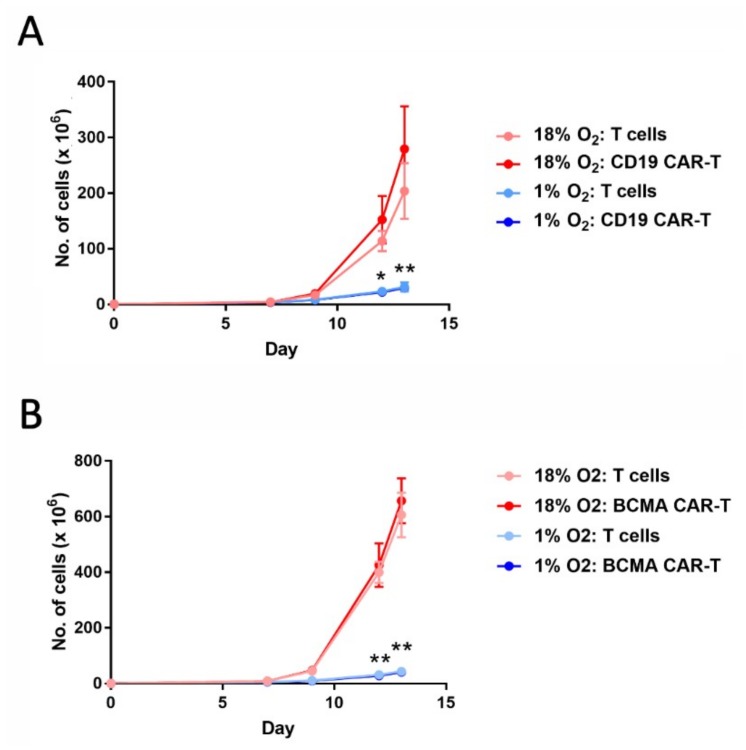
Hypoxia decreases chimeric antigen receptor-T cell (CAR-T cell) expansion. CD19 CAR-T cells (**A**) and B cell maturation antigen (BCMA) CAR-T cells (**B**), along with control T cells, were cultured in an 18% oxygen incubator for the entire 13-day expansion period (red lines), or were cultured in the 18% oxygen incubator for the first 5 days and then in a hypoxia chamber for the remaining 8 days (blue lines). Data-points represent the average and standard error of 4 separate experiments. * *p* = 0.02 (day 12) and ** *p* < 0.001 (day 13) for hypoxic vs. atmospheric CD19 CAR-T cells. ** *p* < 0.001 for hypoxic vs. atmospheric BCMA CAR-T cells.

To determine the effects of hypoxia on CAR-T cell expansion, CD19 CAR-T cells and BCMA CAR-T cells were transferred into a chamber continually maintaining an oxygen level of 1% on day 5 of the expansion period. The hypoxia chamber was placed inside a tissue culture incubator (humidified, with 5% carbon dioxide), and cell expansion was monitored for another eight days. As shown in Figure 1, hypoxia greatly diminished the expansion of both CAR-T cells and control (non-transduced) T cells.

### 2.2. Hypoxia Does Not Affect CAR-T Cell Frequency

**Figure 2:**
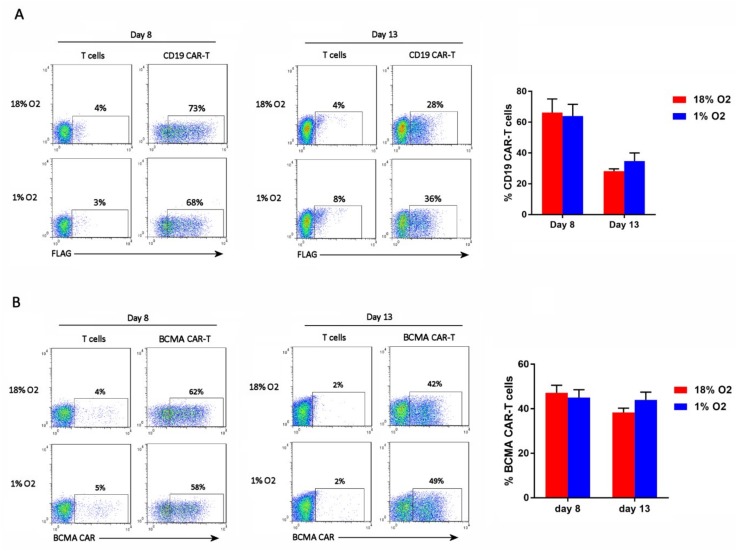
Hypoxia does not affect CAR-T cell frequency. CD19 CAR-T cells (**A**) and BCMA CAR-T cells (**B**) were stained with an anti-FLAG antibody or BCMA protein, respectively. Representative flow cytometry plots showing CAR expression on the X-axis (the Y-axis is an empty channel) are on the left. Charts showing the average and standard error of 4 separate experiments are shown on the right.

The cells were analyzed by flow cytometry on days 8 and 13 of the expansion period for CAR expression. CD19 CAR-T cells were detected with an anti-FLAG antibody, whereas BCMA CAR-T cells were detected with BCMA protein. As shown in Figure 2, hypoxia did not affect the percentage of cells that expressed the CAR (i.e., the CAR-T cell frequency).

### 2.3. Hypoxia Inhibits CAR-T Cell Differentiation

**Figure 3:**
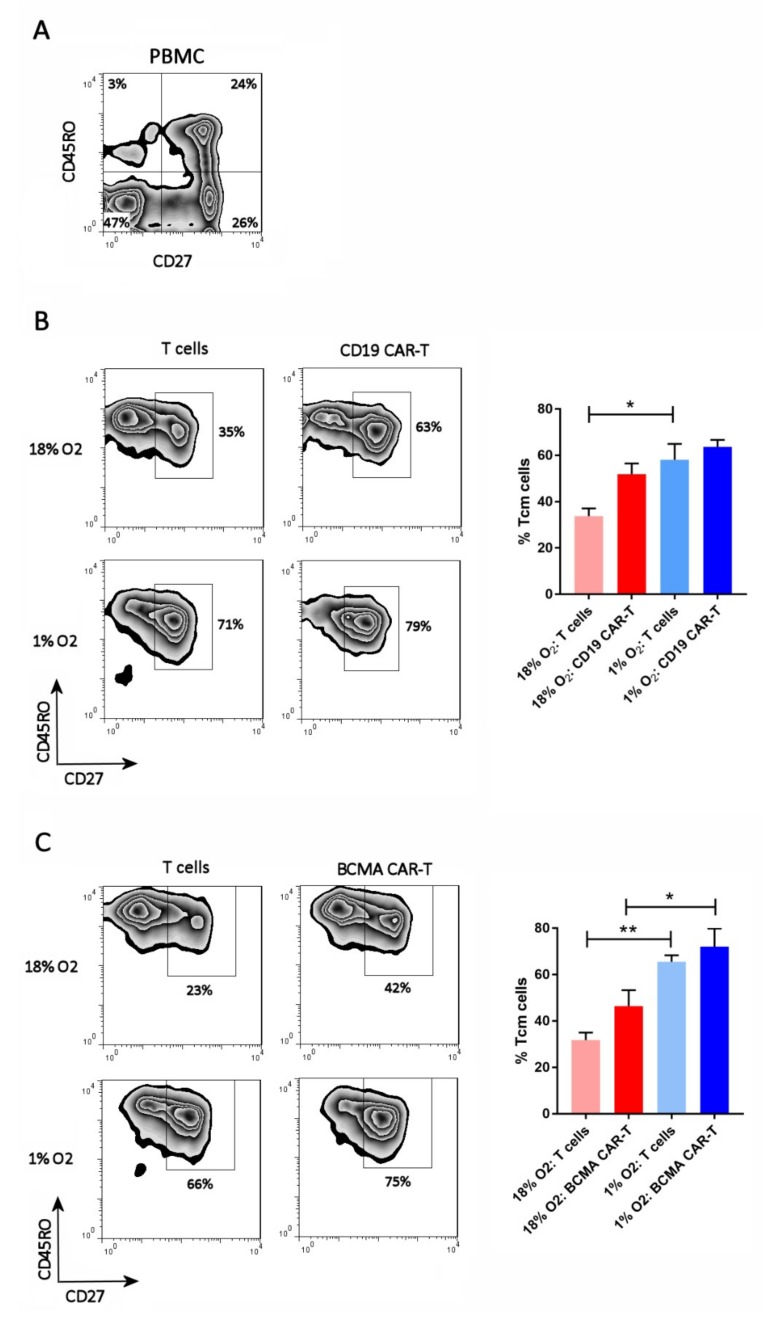
Hypoxia inhibits CAR-T cell differentiation. PBMC (**A**), CD19 CAR-T cells (**B**) and BCMA CAR-T cells (**C**) were stained with antibodies for CD27 and CD45RO. CAR-T cells were first gated using the anti-FLAG antibody or BCMA protein. Representative flow cytometry plots showing CD27 and CD45RO expression are on the left; the CAR-T plots show only the gated CAR-T cells. Charts showing the average and standard error of 4 separate experiments are shown on the right. * *p* < 0.05 and ** *p* < 0.005.

The cells were analyzed by flow cytometry on day 13 of the expansion period for T cell differentiation subsets. Antibodies specific for CD27 and CD45RO were used, as they discriminate the 4 primary subsets (from least to most differentiated): naïve T cells (Tn, CD27^+^CD45RO^−^), central memory T cells (Tcm, CD27^+^CD45RO^+^), effector memory T cells (Tem, CD27^–^CD45RO^+^), and effector T cells (Teff, CD27^–^CD45RO^−^). The FLAG antibody or BCMA protein was included, to identify the CD19 CAR-T cells or BCMA CAR-T cells, respectively (see Figure S1 for the gating strategy). As shown in Figure 3, all of the CAR-T cells and control T cells, in both the 18% oxygen culture and 1% oxygen culture, were memory T cells (CD45RO^+^). Hypoxia caused an increase in the frequency of central memory cells (CD27^+^) in the control T cell cultures and the BCMA CAR-T cell culture, and showed a trend towards doing the same in the CD19 CAR-T cell culture (Figure 3). Hence, the differentiation of Tcm cells into Tem cells was largely impaired in the hypoxic cultures.

### 2.4. Hypoxia Increases the CAR-T Cell CD4:CD8 Ratio

**Figure 4:**
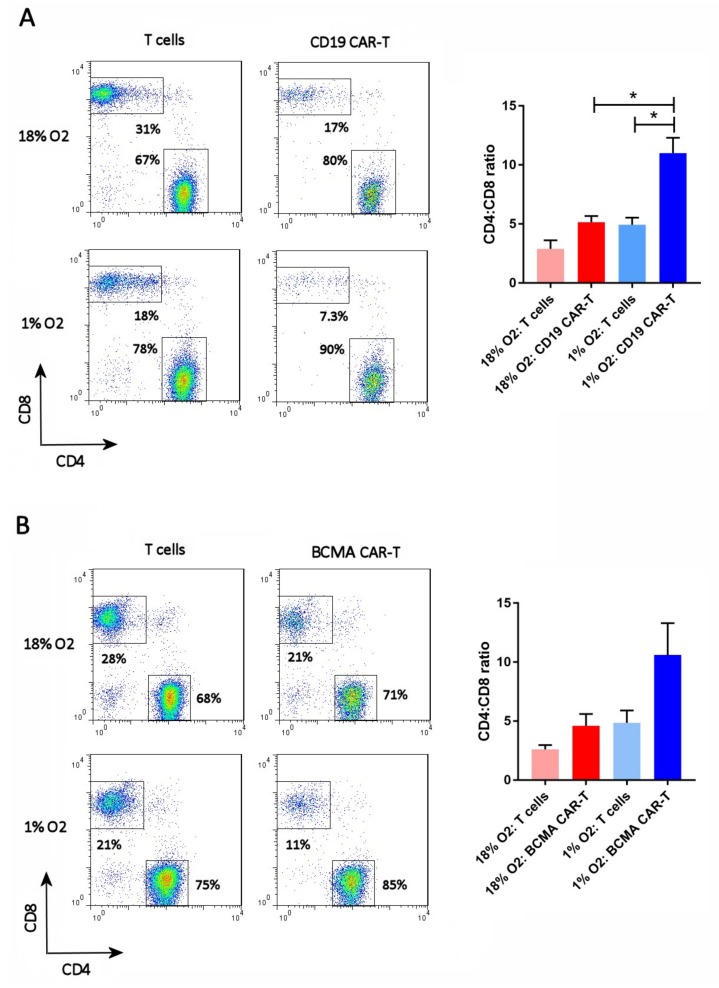
Hypoxia increases the CD4:CD8 ratio. CD19 CAR-T cells (**A**) and BCMA CAR-T cells (**B**) were stained with antibodies for CD27 and CD45RO, along with the anti-FLAG antibody or BCMA protein. Representative flow cytometry plots showing CD27 and CD45RO expression are on the left; the CAR-T plots show only the gated CAR-T cells. Charts showing the average and standard error of 4 separate experiments are shown on the right. * *p* = < 0.05.

The cells were analyzed on day 13 for the ratio of CD4 T cells to CD8 T cells. In normal human PBMC, this ratio is typically 2:1. The FLAG antibody or BCMA protein was included in the staining, to gate on the CD19 CAR-T cells or BCMA CAR-T cells, respectively. As shown in Figure 4, the CD4:CD8 ratio of atmospheric T cells was approximately 2.5:1, whereas the CD4:CD8 ratio of atmospheric CAR-T cells was approximately 5:1. In contrast, the CD4:CD8 ratio of hypoxic T cells was 5:1 and the CD4:CD8 ratio of hypoxic CAR-T cells was 8.6:1 (BCMA CAR-T cells) or 11:1 (CD19 CAR-T cells). Hence, hypoxia increased the CD4:CD8 ratio of both CD19 and BCMA CAR-T cells, and the CAR-T cells themselves had a higher CD4:CD8 ratio than non-transduced T cells.

### 2.5. Hypoxia Does Not Affect CAR-T Cell Cytotoxicity

**Figure 5:**
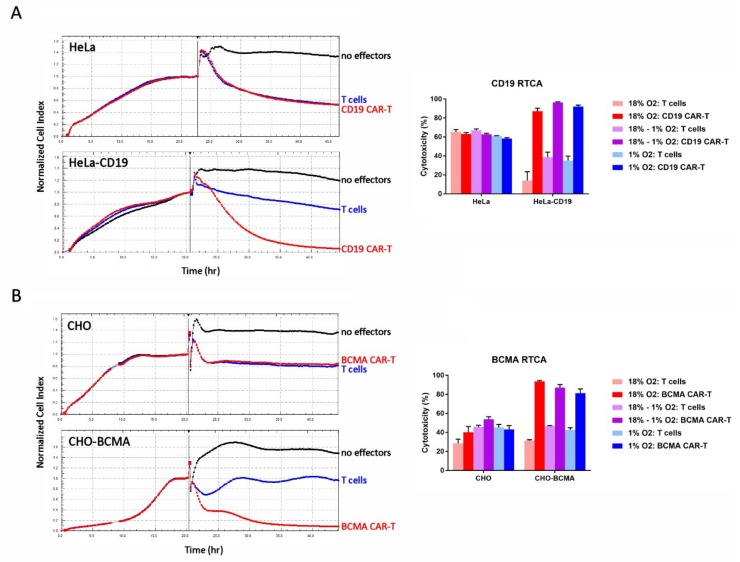
Hypoxia does not affect CAR-T cell cytotoxicity. (**A**) CD19 CAR-T cell RTCA assay. (**B**) BCMA CAR-T cell RTCA assay. Left: HeLa, HeLa-CD19, CHO, and CHO-BCMA cells were monitored overnight as they adhered to the plate and formed a monolayer. The next day, atmospheric CD19 CAR-T cells, BCMA CAR-T cells or control T cells were added to the monolayers at an E:T ratio of 10:1 (vertical bars). The cultures were monitored for approximately 24 more hours. Traces show the average of 3 wells. Right: Cytotoxicity in the RTCA assays was calculated at the end of the assays. Data-points represent the average and standard error of 4 separate experiments.

The cytolytic activity of the cells was evaluated on day 13 using a real-time cellular analysis (RTCA) assay. In this assay, the effector cells (CAR-T cells or control T cells) are added to a monolayer of antigen-positive or antigen-negative target cells and the integrity of the monolayer—determined by its impedance in an electrical field—is monitored over time. If the effector cells kill the target cells, the impedance of the monolayer decreases. As shown in Figure 5, both CD19 CAR-T cells and BCMA CAR-T cells killed cell lines stably expressing CD19 and BCMA, respectively, to a significantly greater extent than the control T cells. In the parental cell lines, the CAR-T cells exhibited cytotoxicity comparable to the control T cells. This CAR-independent cytotoxicity is likely an allogeneic phenomenon, since the effector cells and target cells are not HLA-matched, and does not produce cytokines like IFNγand IL-2. CAR-mediated cytotoxicity did not differ significantly between the three conditions tested: (1) Atmospheric CAR-T cells mixed with atmospheric target cells, (2) atmospheric CAR-T cells mixed with hypoxic target cells in the hypoxia chamber, and (3) hypoxic CAR-T cells mixed with hypoxic target cells in the hypoxia chamber (Figure 5).

### 2.6. Hypoxia Decreases CAR-T Cell Granzyme B and Cytokine Production in Response to Transfected Cell Lines

**Figure 6:**
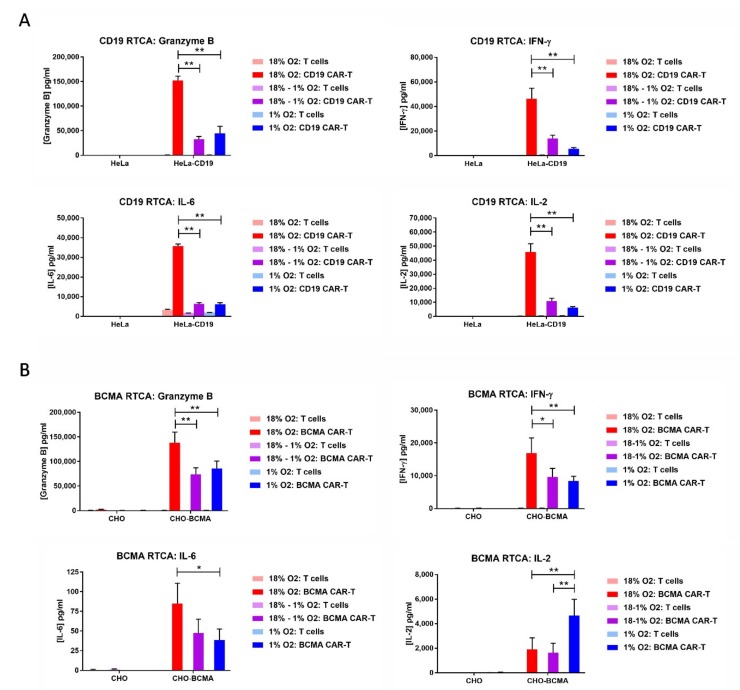
Hypoxia decreases CAR-T cell granzyme B and cytokine production. The media from the CD19 RTCA assay (**A**) and BCMA RTCA assay (**B**) was analyzed by ELISA for the levels of granzyme B, IFN-γ, IL-2 and IL-6. Data-points represent the average and standard error of 2–4 separate experiments. * *p* < 0.05 and ** *p* < 0.005.

The cell culture media from the RTCA assays was analyzed for the levels of the serine protease granzyme B and the cytokines IFN-γ, IL-2 and IL-6. In most cases, the levels of all 4 analytes were substantially decreased when the RTCA assay was performed in 1% oxygen compared to 18% oxygen, regardless of whether the CAR-T cells were originally expanded in 18% oxygen or 1% oxygen (Figure 6). BCMA CAR-T cell production of IL-2 did not follow this pattern, as atmospheric cells cultured in either 18% oxygen or 1% oxygen produced low IL-2 levels and hypoxic cells produced 2.5-fold higher levels. BCMA CAR-T cells produced extremely low levels of IL-6 regardless of oxygen level.

### 2.7. Hypoxia Decreases CAR-T Cell Granzyme B and Cytokine Production in Response to Tumor Cells

**Figure 7:**
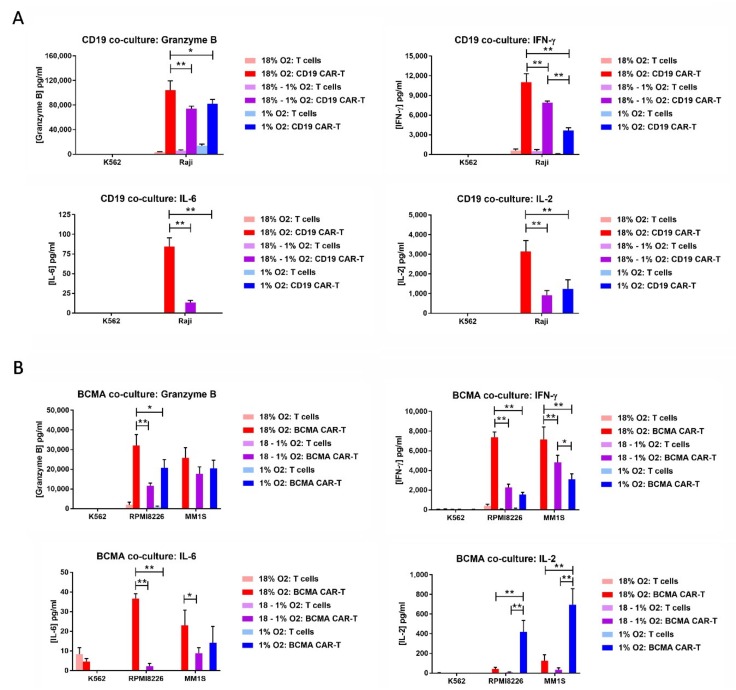
Hypoxia decreases CAR-T cell granzyme B and cytokine production in response to tumor cells. CD19 CAR-T cells or control T cells (**A**) were co-cultured with CD19^+^ Raji cells or CD19^−^ K562 cells. BCMA CAR-T cells or control T cells (**B**) were cultured with BCMA^+^ RPMI8226 cells, BCMA^+^ MM1S cells or BCMA^–^ K562 cells. The medium from the co-cultures was analyzed by ELISA for the levels of granzyme B, IFN-γ, IL-2, and IL-6. Data-points represent the average and standard error of 2-4 separate experiments. * *p* < 0.05 and ** *p* < 0.005.

On day 12 of the expansion period, the CAR-T cells or control T cells were co-cultured overnight at a 10:1 E:T ratio with hematopoietic cell lines endogenously expressing or lacking CD19 or BCMA. CD19 CAR-T cells were cultured with B lymphoma Raji cells, which express CD19, or with myelogenous leukemia K562 cells, which do not express CD19. BCMA CAR-T cells were cultured with multiple myeloma RPMI8226 cells or MM1S cells, both of which express BCMA, or with K562 cells, which do not express BCMA. As before, three conditions were tested: (1) Atmospheric CAR-T cells mixed with atmospheric tumor cells in the normal incubator, (2) atmospheric CAR-T cells mixed with hypoxic tumor cells in the hypoxia chamber, and (3) hypoxic CAR-T cells mixed with hypoxic tumor cells in the hypoxia chamber. The next day, the medium in the co-cultures was assayed for the levels of granzyme B, IFN-γ, IL-2, and IL-6. For both CD19 CAR-T cells and BCMA CAR-T cells, the levels of granzyme B, IFN-γ and IL-6 were lower in the co-cultures incubated at 1% oxygen than the co-cultures incubated at 18% oxygen, regardless of whether the CAR-T cells were originally atmospheric or hypoxic (Figure 7). This was also true for IL-2 production by CD19 CAR-T cells, but not BCMA CAR-T cells. Hypoxic BCMA CAR-T cells produced low levels of IL-2, whereas atmospheric BCMA CAR-T cells produced very low levels of IL-2. Both CD19 CAR-T cells and BCMA CAR-T cells produced very low levels of IL-6.

### 2.8. Hypoxia Does Not Affect CAR-T Cell PD-1 Upregulation

**Figure 8:**
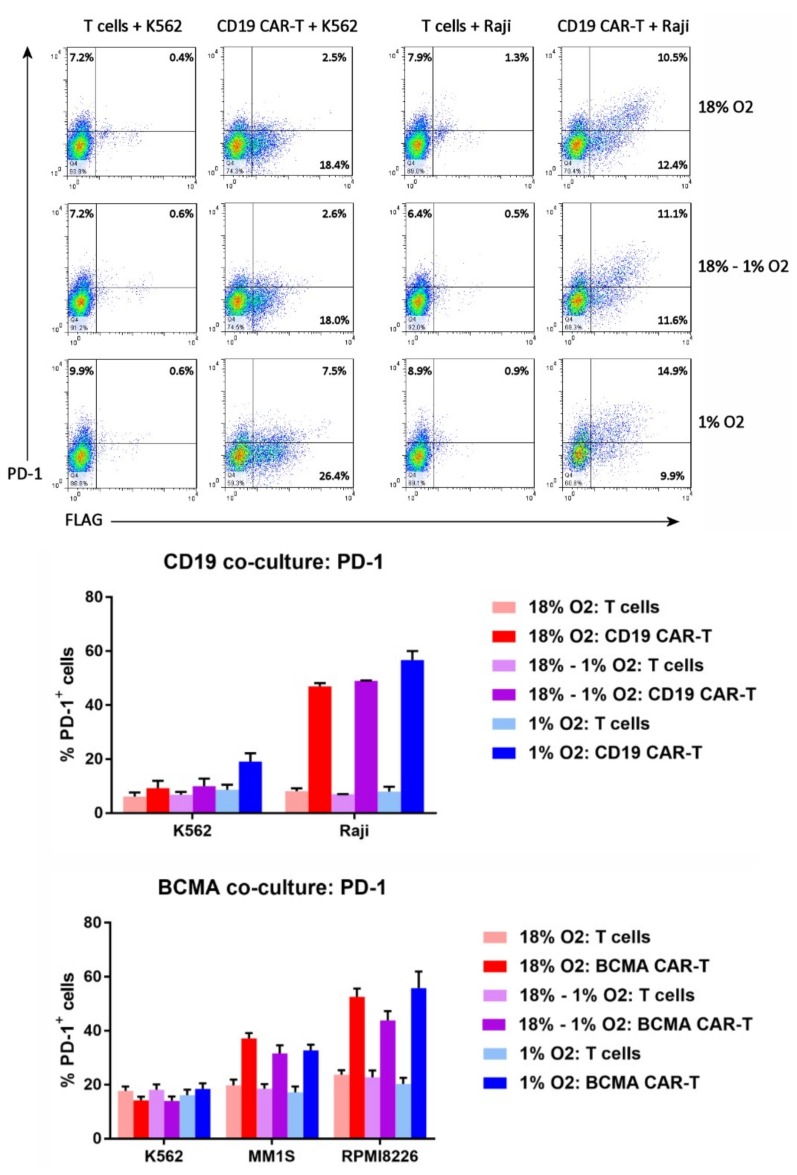
Hypoxia does not affect CAR-T cell PD-1 upregulation. Top: the cells from the CD19 CAR-T cell co-cultures were analyzed by flow cytometry for FLAG staining (i.e., CD19 CAR expression) vs. PD-1 expression. Bottom: the percentages of CD19 CAR-T cells, BCMA CAR-T cells, or control T cells expressing PD-1 were plotted; data-points represent the average and standard error of 2–4 separate experiments.

Since T cells upregulate PD-1 upon tumor cell recognition, we wanted to know whether PD-1 upregulation is altered in hypoxic settings; if hypoxia amplifies PD-1 upregulation, higher levels of checkpoint protein inhibitors like Kymriah might be required for activity in solid tumors. Therefore, the cells from the co-cultures were analyzed by flow cytometry for expression of PD-1. The FLAG antibody or BCMA protein was included in the staining, to gate on the CD19 CAR-T cells or BCMA CAR-T cells, respectively. As shown in Figure 8, PD-1 was expressed on a significantly higher percentage of CAR-T cells than control T cells when the cells were co-cultured with antigen-positive tumor cells (Raji, MM1S or RPMI8226). In contrast, PD-1 expression was comparable between CAR-T cells and control T cells when the cells were cultured with antigen-negative K562 cells. Importantly, the frequency of antigen-mediated PD-1 upregulation on CAR-T cells was not affected by the level of oxygen during the expansion period or during the co-culture.

### 2.9. CAR-T Cell Expansion in 5% Oxygen Results in Greater Cytotoxicity and Decreased IFN-γ/IL-2 Production

**Figure 9:**
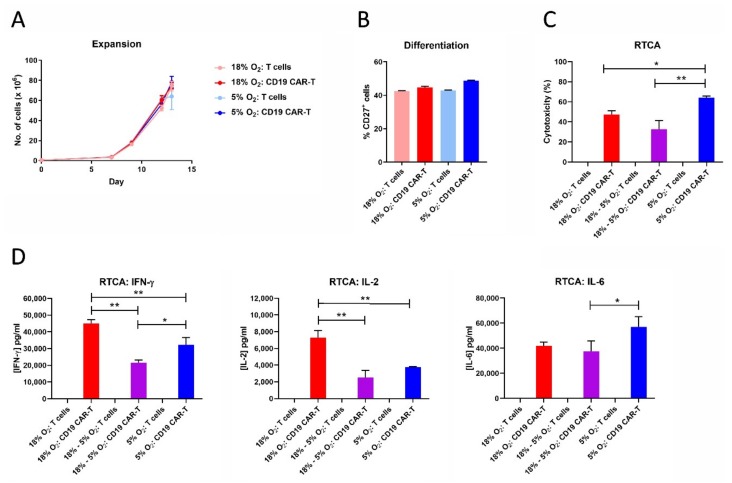
CAR-T cell expansion in 5% oxygen results in greater cytotoxicity and decreased IFN-γ/IL-2 production. CD19 CAR-T cells and control T cells expanded in 18% oxygen or 5% oxygen were analyzed for cell expansion (**A**), differentiation (**B**), cytotoxicity (**C**), and cytokine production during the RTCA assay (**D**). Data points indicate averages of 2–3 replicates; * *p* < 0.05 and ** *p* < 0.005.

Since hematological cancers reside partly in the bloodstream, which is more oxygenated than solid tumors, we tested the effect of 5% oxygen on CD19 CAR-T cell expansion, differentiation, cytotoxicity and cytokine production. Unlike 1% oxygen, 5% oxygen did not impair CAR-T cell expansion or CAR-T cell differentiation (Figure 9). Interestingly, CAR-T cells expanded in 5% oxygen were actually more cytotoxic against HeLa-CD19 target cells than were CAR-T cells expanded in 18% oxygen, even if the latter cells were assayed in 5% oxygen. Despite the increased cytotoxicity, CAR-T cells expanded and assayed in 5% oxygen produced lower levels of IFN-γ and IL-2 than CAR-T cells expanded and assayed in 18% oxygen. In addition, CAR-T cells expanded in 18% oxygen produced less IFN-γ and IL-2 when assayed in 5% oxygen—similar to when the cells were assayed in 1% oxygen (Figure 6 and Figure 7). In contrast to IFN-γ and IL-2, IL-6 levels were not decreased by expanding or assaying the CD19 CAR-T cells in 5% oxygen. These data show that 1% oxygen has much stronger effect on CAR-T cell functions than 5% oxygen.

## 3. Discussion

In this study we compared CAR-T cells expanded under atmospheric (18%) oxygen levels to CAR-T cells expanded under hypoxic (1%) oxygen levels. Expansion of hypoxic CD19 CAR-T cells and BCMA CAR-T cells, as well as control T cells, was impaired 10–20-fold over the eight-day expansion period. The impairment was not due to altered T cell activation or lentiviral transduction, since these events occurred in the 5-day period before expansion, when all cultures were kept in atmospheric oxygen. Previous studies of non-CAR-T cells found that hypoxia reduced proliferation rates and increased apoptosis rates [19,20,21,22,23,24]. However, the mechanisms by which hypoxia affects proliferation and apoptosis are not clear. Prior studies have indicated that hypoxia is associated with alterations in Kv1.3 potassium channel activity that impair CD3ζ-mediated Ca^2+^ signaling [25], and that IL-2 signaling might also be impaired [26]. Hypoxia inducible factor 1α may be involved, as it can interact with MCM replication proteins to regulate cell cycle progression [27]. Clearly, this is an important area requiring a future detailed, comprehensive analysis.

Although hypoxia inhibited CAR-T cell expansion, the frequency of CAR-T cells in the cultures was not affected by hypoxia. This suggests that hypoxia affects the expansion of CAR-T cells and non-transduced T cells equally, and does not cause CAR down-regulation. Hypoxia did impair the differentiation of central memory BCMA CAR-T cells into effector memory BCMA CAR-T cells, and showed a trend towards doing the same to CD19 CAR-T cells. This suggests that differentiation of CAR-T cells is oxygen-dependent, which is consistent with prior studies on non-CAR-T cells [19,21,22,23]. The impaired differentiation of CAR-Tcm cells into CAR-Tem cells might actually be favorable therapeutically, since Tcm cells exhibit enhanced persistence after adoptive transfer [28]. Since culture of CD19 CAR-T cells in 5% oxygen did not affect their expansion or differentiation, oxygen levels below 5% are required for the reduction in differentiation. In addition, initial experiments indicate that hypoxia skews the balance of CAR-T cell functional subsets from a Th1/Tc1-dominated composition to a Treg-dominated composition.

Hypoxia also increased the CD4:CD8 ratio of the CAR-T cells, suggesting that CD4^+^ CAR-T cell expansion is less oxygen-dependent than CD8^+^ CAR-T cell expansion. Previous studies of non-CAR-T cells also found hypoxia-mediated increases in the CD4:CD8 ratio [19]. In fact, it is possible that the hypoxia-mediated increased CD4:CD8 ratio and decreased differentiation we observed might be related; perhaps CD4^+^ CAR-T cells differentiate more slowly than CD8^+^ CAR-T cells in culture. In addition, we observed that, in both atmospheric and hypoxic cultures, both CD19 CAR-T cells and BCMA CAR-T cells had a higher CD4:CD8 ratio than control T cells. This suggests either that CD4^+^T cells are transduced by the CAR lentivirus more readily than CD8^+^ T cells, or that CAR expression impacts CD4^+^T cell expansion less than CD8^+^ T cell expansion. These important questions should be answered in our next study, when we analyze CAR-T cells generated from isolated CD4^+^ T cells and CD8^+^ T cells.

Functionally, CAR-T cells expanded under hypoxia were not impaired in their ability to kill cells transfected to stably express the target antigen, consistent with prior studies on non-CAR-T cells [19,24,29]. The equivalent cytotoxicity coupled with the increased CD4:CD8 ratio indicates that CD4^+^ CAR-T cells are cytotoxic in vitro [30]. In the clinic, a 1:1 mixture of separate CD4^+^ CAR-T cells and CD8^+^ CAR-T cells was found to be highly efficacious in adult B-ALL patients [31]. However, several recent studies indicate that a CD4:CD8 ratio greater than 1:1 might be beneficial, especially in solid tumors. Wang et al found that IL-13 receptor α2-specific CD4^+^ CAR-T cells exhibited long-term cytotoxicity against primary glioblastoma cells, whereas CD8^+^ CAR-T cells exhibited short-term cytotoxicity but became exhausted, permitting tumor relapse [32]. The CD4^+^ CAR-T cells had significant upregulation of genes responsible for stem cell renewal and memory function such as WNT9B, WNT9A, AXIN2, LEF, TWIST1, ALDH1A3 and EGFR [32]; perhaps these genes helped CD4^+^ CAR-T cells overcome the hypoxia-mediated inhibition of expansion we observed, increasing the CD4:CD8 ratio. Analysis of CAR-T cell products from GBM patients indicated that the products with decreased CD4:CD8 ratios demonstrated decreased cytotoxicity [32]. In multiple myeloma, Cohen et al showed that a higher CD4:CD8 T cell ratio in the leukapheresis product was associated with greater CAR-T cell expansion in the patient and with a greater therapeutic response [33]. Interestingly, since the CAR-T cells we expanded in 5% oxygen were more cytotoxic than the CAR-T cells expanded in 18% oxygen, it is possible that the clinical efficacy of CAR-T cells could be improved by expanding them in 5% oxygen. 

Despite their high cytolytic activity, hypoxic CAR-T cells produced relatively low levels of granzyme B, IFN-γ, IL-2, and IL-6 in response to transfected cells and tumor cell lines endogenously expressing the target antigen. This is consistent with prior studies on non-CAR-T cells [19,23,24]. Interestingly, this effect also occurred when atmospheric CAR-T cells were cultured with hypoxic target cells. The decreased levels were not due to decreased target expression on the transfected cells and tumor cells; flow cytometric analysis indicated that CD19 and BCMA expression levels on the target cells were not altered in 1% oxygen. Thus, decreased granzyme B and cytokine production occurs in the hypoxic setting, even if the CAR-T cells are expanded in atmospheric oxygen. This is the case in vivo, where CAR-T cells in the bloodstream move out of the capillaries and into the hypoxic tumor or bone marrow. Next-generation CAR-T cells with increased production of cytokines might be needed to overcome this hypoxia-mediated effect.

Lastly, hypoxia did not affect checkpoint protein PD-1 upregulation. Hence, PD-1 blocking antibodies should be as active in solid tumors as the antibodies are in non-hypoxic settings. In fact, next-generation CAR-T cells with down-regulation of the PD-1 pathway exhibit enhanced activity in solid tumors [7,34,35]. CRISPR/Cas9-mediated disruption of the PD-1 pathway was actually more effective than adding a PD-1 neutralizing antibody in enhancing CAR-T cytotoxicity against solid tumors [35].

In summary, this is the first report to describe the effects of hypoxia on CAR-T cells. The effects we observed were consistent between CD19 CAR-T cells and BCMA CAR-T cells, indicating that the effects are CAR-independent and likely to apply to other targets. These data are critical for clinical studies because the success of CAR-T cell therapy depends on multiple parameters affected by hypoxia, including CAR-T cell expansion, CAR-T cell functional activity, and CAR-T cell differentiation/maturation status [36,37]. In particular, the less differentiated phenotype of CAR-T cells was found to preferable for expansion and persistence in patients, and adequate number of CD4^+^ CAR-T cells and CD8^+^ CAR T cells in the manufactured CAR-T product was obtained without pre-selection of T cell subsets [36]. Our experiments also provide baseline data important for the design of hypoxia-resistant next-generation CAR-T cells. Analyzing the other aspects of the solid tumor microenvironment on CAR-T cell expansion, differentiation, cytotoxicity, cytokine production, and checkpoint protein expression will be critical for developing successful CAR-T cell therapies against solid tumors in the future.

## 4. Materials and Methods 

### 4.1. Cells

HeLa cells were purchased from the ATCC (Manassas, VA, USA) and cultured in DMEM (GE Healthcare, Chicago, IL, USA) containing 10% FBS (Lonza, Walkersville, MD, USA). K562, Raji, MM1S and RPMI8226 cells were purchased from the ATCC and cultured in RPMI-1640 medium (Thermo Fisher, Waltham, MA, USA) containing 10% FBS. CHO-CD22 and CHO-BCMA cells were purchased from BPS Bioscience (San Diego, CA, USA) and cultured in Ham’s F-12K medium (Thermo Fisher) containing 10% FBS and 1 mg/mL geneticin (Thermo Fisher). HeLa-CD19 cells were generated in our laboratory [16] and cultured in DMEM containing 10% FBS and 1 uM puromycin (Thermo Fisher). Human peripheral blood mononuclear cells (PBMC) were isolated from LRS chambers (Stanford Blood Center, Palo Alto, CA, USA) by density sedimentation over Ficoll-Paque (GE Healthcare). HEK293FT cells were a gift from AlStem (Richmond, CA, USA) and were cultured in DMEM containing 10% FBS.

### 4.2. Generation of CAR-Encoding Lentivirus

Ten million growth-arrested HEK293FT cells were seeded into T75 flasks and cultured overnight, then transfected with the pPACKH1 Lentivector Packaging mix (System Biosciences, Palo Alto, CA, USA) and 10 μg of either the CD19-FLAG [16] or BCMA 4C8A [18] lentiviral vector using the CalPhos Transfection Kit (Takara, Mountain View, CA, USA). The next day the medium was replaced with fresh medium, and 48 h later the lentivirus-containing medium was collected. The medium was cleared of cell debris by centrifugation at 2100× *g* for 30 min. The virus particles were collected by centrifugation at 110,000× *g* for 100 min, suspended in AIM V medium (Thermo Fisher), aliquoted and frozen at −80 °C, as described [16,37,38,39].

### 4.3. Generation and Expansion of CAR-T Cells

Treated 24-well plates were incubated at 37 °C for 2 hours with PBS containing 0.1 μg/mL anti-CD3 clone OKT3 (Biolegend, San Diego, CA, USA) and 0.1 μg/mL anti-CD28 clone CD28.2 (Thermo Fisher). PBMC were suspended at 1 × 10^6^ cells/mL in AIM V medium containing 10% FBS and 10 ng/ml of IL-2 (Thermo Fisher). The wells were rinsed with PBS, then 0.5 mL of PBMC were added per well. The next day, 20 uL of lentivirus was added to the cells, along with 5 μg/mL of DEAE-dextran (Sigma, St. Louis, MO, USA). The day after that (day 2), another 20 ul of lentivirus was added to the cells. Three days later, half of the cells were transferred into a humidified C chamber (Biospherix, Parish, NY, USA) set to 5% carbon dioxide and either 5% oxygen or 1% oxygen. As the T cells proliferated over the next 8 days, the cells in the normal incubator and hypoxia chamber were counted every 2–3 days and fresh medium (equilibrated overnight in the hypoxia chamber for the hypoxic cultures) with IL-2 was added to the cultures to maintain the proper cell density.

### 4.4. Flow Cytometry

To measure CAR expression, cells were first suspended in 100 μL of cold buffer (PBS containing 0.5% BSA and 2 mM EDTA) supplemented with 2 μg of goat IgG (Jackson Immunoresearch, West Grove, PA, USA) and incubated on ice. CD19-FLAG CAR-T cells were stained with 2 μL of PE- or Alexa Fluor 488-conjugated anti-FLAG (Biolegend), whereas BCMA CAR-T cells were first stained with 0.4 μg of BCMA-huFc protein (Acro Biosystems, Newark, DE, USA) and then stained with 1 μL of PE- or Alexa Fluor 488-conjugated goat anti-human IgG (Jackson Immunoresearch). Cells were co-stained with either FITC-conjugated anti-CD4 and APC-conjugated anti-CD8; PE-conjugated anti-CD27 and APC-conjugated CD45RO; or APC-conjugated anti-PD-1 (all from Biolegend). Dead cells were identified with 7-aminoactinomycin D (7-AAD, BioLegend). The cells were rinsed with 3 mL of buffer, then suspended in buffer and acquired on a FACSCalibur (BD Biosciences, San Jose, CA, USA). Gating strategies are shown in Supplemental Figure S1.

### 4.5. Cytotoxicity Assay

Adherent target cells were seeded into 96-well E-plates (Acea Biosciences, San Diego, CA) at 1.5 × 10^4^ cells per well (HeLa-CD19), 3 × 10^4^ cells per well (HeLa), or 4 × 10^4^ cells per well (CHO-CD22, CHO-BCMA), and monitored in the normal incubator or hypoxia chamber overnight with the xCELLigence impedance-based RTCA system (Acea Biosciences). The next day, the medium was removed and replaced with normal or equilibrated RPMI-1640 medium containing 10% FBS ± CAR-T cells or non-transduced T cells at an E:T ratio of 10:1, in triplicate. The cells in the E-plates were monitored for another 20–24 h with the RTCA system, and impedance (normalized to the time of effector cell addition) was plotted over time. Cytotoxicity was calculated as the percentage (X − Y) × 100/X, where X = normalized impedance of target cells without effector cells and Y = normalized impedance of target cells with effector cells. For hypoxic RTCA assays, target cells were cultured in the hypoxia chamber for three days before use.

### 4.6. Cytokine and PD-1 Induction Assay

Effector cells (CAR-T cells or non-transduced T cells) were co-cultured overnight with target cells (Raji, K562, MM1S or RPMI8226 cells) at a 10:1 E:T ratio in normal or equilibrated RPMI-1640 medium containing 10% FBS, in duplicate or triplicate. The next day, the cultures were centrifuged at 300 g, the supernatants were transferred to new tubes and frozen, and the cells were suspended in FACS buffer and analyzed by flow cytometry for expression of the CAR and PD-1. Culture supernatants were later thawed and analyzed by ELISA for the levels of IFN-γ, IL-2, IL-6, and Granzyme B according to the manufacturer’s protocols (R&D Systems, Minneapolis, MN, USA). For hypoxic co-cultures, tumor cells were cultured in the hypoxia chamber for three days before use.

### 4.7. Statistical Analysis

Data were analyzed and plotted with Prism software v8.1.1 (GraphPad, San Diego, CA, USA). Comparisons between two groups were performed by unpaired Student’s *t* test, and comparisons between three or more groups were performed by one-way or two-way ANOVA with Tukey’s post-hoc test. 

## 5. Conclusions

CD19-specifc CAR-T cells and BCMA-specific CAR-T cells were selectively affected by exposure to hypoxia (1% oxygen). CAR expression, CAR-T cell cytolytic activity and target antigen-induced PD-1 upregulation were not affected by hypoxia. However, CAR-T cell expansion, differentiation, CD8:CD4 ratio, and production of granzyme B and cytokines IFN-γ, IL-2, and IL-6 were all significantly decreased by hypoxia. These effects may underlie the failure of CAR-T cells to eradicate solid tumors in some patients and point to areas in which CAR-T cells may be modified for future clinical studies.

## Supplementary Materials

The following are available online at https://www.mdpi.com/2072-6694/11/5/602/s1, Figure S1: Gating strategies used to identify cells by flow cytometry.
