# Supplementary material for: Hypoxia Selectively Impairs CAR-T Cells In Vitro
Source: Cancers (Basel). 2019 Apr 30;11(5):602. doi: 10.3390/cancers11050602 (PMC6562712; doi:10.3390/cancers11050602)
Supplement: Supplementary file 1 [file cancers-11-00602-s001.pdf]

Supplementary Materials: Hypoxia Selectively Impairs CAR-T Cells In Vitro

Robert Berahovich, Xianghong Liu, Hua Zhou, Elias Tsadik, Shirley Xu, Vita Golubovskaya and Lijun Wu

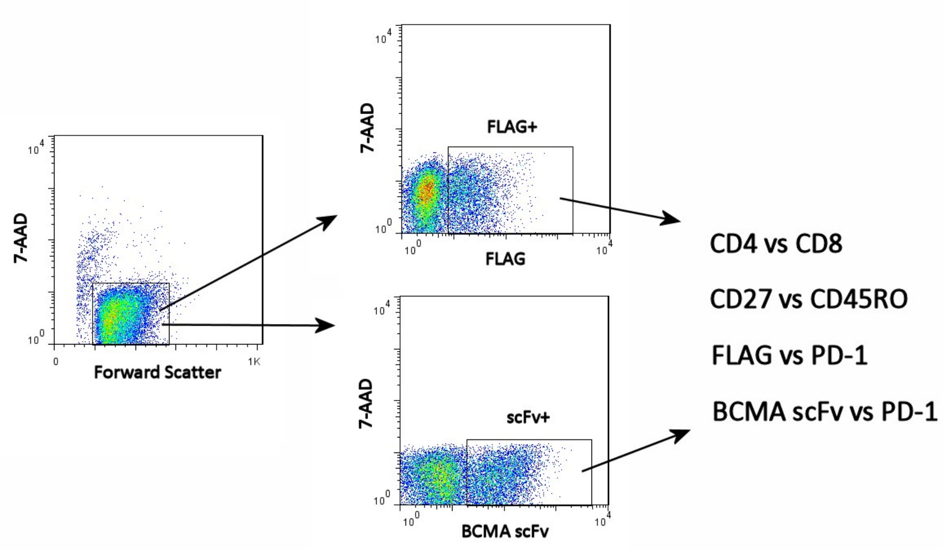

Figure S1. Gating strategies used to identify cells by flow cytometry. Cells were first analyzed by forward light scatter versus 7-AAD staining (left plot), and a gate was drawn around the live (i.e., 7-AAD-negative) cells. Next, the live cells were analyzed for binding to the FLAG antibody (CD19 CAR-T cells, middle top plot) or to the BCMA protein (BCMA CAR-T cells, middle bottom plot). Gates were drawn around the stained cells, and the gated cells (CAR-T cells) were analyzed for CD4 vs CD8 antibody staining (Figure 4) and CD27 vs CD45RO antibody staining (Figure 3). In the case of control T cells, all the live cells were analyzed for CD4 vs CD8 and CD27 vs CD45RO. For PD-1 expression, all the live cells were analyzed for PD-1 antibody binding vs FLAG antibody binding (CD19 CAR-T cells) or BCMA protein binding (BCMA CAR-T cells).

© 2019 by the authors. Licensee MDPI, Basel, Switzerland. This article is an open access article distributed under the terms and conditions of the Creative Commons Attribution (CC BY) license (http://creativecommons.org/licenses/by/4.0/).
